# Remdesivir Use in Low Weight, Premature, and Renally Impaired Infants With SARS-CoV-2 Infection in Sheikh Khalifa Medical City, UAE: Case Series

**DOI:** 10.7759/cureus.33591

**Published:** 2023-01-10

**Authors:** Wadha Mohammed, Nada Al Saqqa, Ayanbanke Ayanleke

**Affiliations:** 1 Clinical Pharmacy, Abu Dhabi Health Services Co (SEHA), Abu Dhabi, ARE

**Keywords:** liver function tests (lfts), kidney disease, remdesivir, premature infants, covid 19

## Abstract

Remdesivir possesses in vitro inhibitory effect against severe acute respiratory syndrome coronavirus 2 and the Middle East respiratory syndrome. It works by inhibiting severe acute respiratory syndrome coronavirus 2 RNA-dependent RNA polymerase that is essential for viral replication. Remdesivir is approved by Food and Drug Administration for treating COVID-19 in hospitalized adult and pediatric patients aged 28 days and more and weighing 3 kg and more. This case series is describing two cases of low-weight, premature, and renally impaired infants where Remdesivir is used in Sheikh Khalifa Medical City pediatric intensive care unit. Upon completion of the Remdesivir course of treatment, there were no Remdesivir-related adverse outcomes noted in the two cases. Remdesivir was tolerated by both patients. However, clinical improvement and measurement of safety and efficacy will require further randomized, placebo-controlled trials.

## Introduction

Children represent a minority of total COVID-19 cases. However, studies have reported severe disease and death in pediatric populations [1]. Although serological evidence of COVID-19 infection is evident in certain pediatric patients, coronavirus has not been detected by Polymerase Chain Reaction (PCR) on nasal swabs in the majority of those children [2]. Moreover, treatment in pediatric patients needs to be individualized after infectious disease physicians and concerned specialty consultations.

Despite the lack of data on the pediatric population, there are a variety of therapeutic options that can be used, such as immunomodulators like steroids and immunostimulants like antiviral medications, including Remdesivir and Favipiravir [3]. Furthermore, immunomodulators such as Anakinra (IL-1 receptor antagonist), Infliximab (monoclonal antibody), Tocilizumab (IL-6 receptor antibody), and intravenous immunoglobulin may also be used [2].

Specifically speaking about Remdesivir, it is an adenosine analog nucleotide prodrug that is administered intravenously [4]. Remdesivir is an anti-Severe Acute Respiratory Syndrome Coronavirus (SARS-CoV) inhibitor and anti-Middle East Respiratory Syndrome (MERS-CoV) RNA-dependent RNA polymerase inhibitor. Due to its capacity to block SARS-CoV-2 in vitro, it was recognized early on as a possible therapeutic approach for COVID-19 [5].

Remdesivir was discovered to hasten recovery from severe COVID-19 and has shown promising outcomes in treating adults with COVID-19 infection [1,6,7]. Remdesivir was reported as potentially improving COVID-19 outcomes and reducing the burden on healthcare resources during the pandemic. Remdesivir use in pediatric patients demonstrated positive clinical outcomes without any observed side effects; however, limited data have been reported to date in children with underweight or renal impairment [1].

The Food and Drug Administration (FDA) has approved its use in treating COVID-19 in hospitalized adult and pediatric patients aged 28 days and more and weighing 3 kg and more. However, it is important to note that the United State FDA has not authorized the use of Remdesivir in children who weigh less than 3 kg [4].

Since clinical trials of Remdesivir in children with COVID-19 are urgently needed to assess the safety and tolerability in underweight or renally impaired patients, we are reporting two of those cases, admitted to Sheikh Khalifa Medical City (SKMC), Abu Dhabi, UAE. For this study, Institutional Review Board approval was obtained.

## Case presentation

For pediatric patients with normal kidney function, the advised Remdesivir dosage is shown in Table 1 [6].

**Table 1:** Remdesivir dosing in the pediatric population

Population	Medication	Route	Dose
Infants and Children 3 kg to < 40 kg	Lyophilized powder ONLY	IV Loading	5 mg/kg/dose on day 1, followed by 2.5 mg/kg/dose once daily
Infants and Children ≥40 kg	Lyophilized powder or solution for injection	IV Loading	200 mg on day 1, followed by 100 mg once daily

Case 1

The patient is a three-week-old female infant with a weight of 3.1 kg at the time of presentation. A full-term infant with antenatal diagnosis of congenital heart disease and post-natal diagnosis of double outlet right ventricle with tetralogy of Fallot physiology left pulmonary artery (LPA) originating from patent ductus arteriosus (PDA). She was electively admitted in 2021 to the cardiac PICU for a cardiac procedure. On the second day of admission, the patient had a sudden cardiovascular collapse with severe metabolic/lactic acidosis, severe low cardiac output syndrome (LCOS), hypotension that required high vasopressors support, and developed multi-organ dysfunction syndrome (MODS) with severe coagulopathy. The patient was started on extracorporeal membrane oxygenation (ECMO) and continuous renal replacement therapy (CRRT). COVID-19 PCR was positive on day 10 of admission, and it was decided to start her on Remdesivir based on the PICU team and ID. When Remdesivir was started, the infant was on ECMO and peritoneal dialysis. Remdesivir was administered as a loading dose of 5 mg/kg on day one and 2.5 mg/kg every day for the next eight days. COVID-19 PCR became negative two days after starting Remdesivir, and the patient was transferred back to the cardiac PICU for continuity of care after finishing the course of Remdesivir. There was no noted adverse outcome during or after Remdesivir treatment. Tables 2, 3 present laboratory characteristics and core investigations before and following the Remdesivir administration.

**Table 2:** Laboratory features and core investigations

Laboratory feature	Result	Normal result
Serum creatinine	150 micromol/L	15-37 micromol/L
C-reactive protein	(CRP) 1.98 mg/l	< 5 mg/l
Procalcitonin	0.81 ng/ml	< 0.5 ng/ml
Lactate dehydrogenase (LDH)	728 IU/l	135-214 IU/l
Ferritin	158 mcg/l	33-835 mcg/l
Allbumin	25 g/L	35-52 g/L
Bilirubin total	50.5 micromol/L	< 17 micromol/L
Bilirubin direct	39.1 micromol/L	< 10 micromol/L
Alkaline phosphate	68 IU/L	35-449 IU/L
Aspartate aminotransferase (AST)	77 IU/L	< 63 IU/L
Alanine aminotransferase (ALT)	34 IU/L	< 29 IU/L
White blood cells (WBC)	13.3 x10 ^9/L	6-17.5 x10 ^9/L
Hemoglobin (Hgb)	100 g/L	134-198 g/L
Platelet	74 x10 ^9/L	140-400 x10 ^9/L
Echocardiogram (Echo) PDA	Tiny Pericardial effusion at the apex	
Sputum culture	No growth	No growth
Wound culture	No growth	No growth
Blood culture	No growth	No growth

**Table 3:** Labs after completing 8 days of Remdesivir (Patient 1)

Laboratory feature	Result	Normal result
↑ Serum creatinine	159 micromol/L	15-37 micromol/L
↓ Albumin	24 g/L	35-52 g/L
↓ AST	35 IU/L	< 63 IU/L
↓ ALT	25 IU/L	< 29 IU/L
↑ Total bilirubin	54 micronol/L	< 17 micromol/L
↓ Alkaline phosphate	46 IU/L	35-449 IU/L
↑ Direct bilirubin	43.1 micromol/L	< 5 micromol/L
↓ LDH	517 IU/l	135-214 IU/l
↑ Ferritin	1247 mcg/L	33-835 mcg/L
First COVID test (2 days after starting Rremdesivir)	Negative	Negative
Second COVID test (4 days after starting Rremdesivir)	Negative	Negative

Case 2

Three-month-old male infant, weight at the presentation of 1.69 kg. The child was born at 32 weeks of gestation and was admitted to the neonatal intensive care unit (NICU) for one month due to a severely low body weight of 1300 grams, then discharged on day 26 of life. At three months of age, the child presented to the emergency with vomiting, difficulty breathing, and a history of COVID-19 exposure, as the mother was positive. On presentation, the child was afebrile, with tachypnea, tachycardia, and low oxygen saturation of 78% and FiO2 of 25%. COVID-19 PCR was done; however, the first result was negative. The infant was admitted to PICU and was on a high-flow nasal cannula (HFNC). The patient was started on antibiotics for possible aspiration pneumonia. On the following day of admission, the second COVID-19 PCR result was positive. The patient was considered a high-risk baby due to premature birth and a family history of immunodeficiency.

The chest X-ray revealed right upper and middle zone infiltrates, and the patient's respiratory effort also deteriorated. The patient was put on Remdesivir following approval from the Multidisciplinary Team (MDT), which included a pediatric infectious disease (ID) and PICU clinical pharmacist. It was decided to start with a Remdesivir loading dose of 5 mg/kg on day one, followed by a maintenance dose of 2.5 mg/kg for a total of 10 days.

The patient additionally got 10 days of antibiotic treatment for bacterial pneumonia (piperacillin-tazobactam, then was switched to ceftriaxone). Below are laboratory characteristics and main investigations (Table 4).

**Table 4:** Laboratory features and core investigations

Laboratory feature	Result	Normal result
Renal function- serum creatinine	18 micromol/L	15-37 micromol/L
Coagulation profile	Normal	
C-reactive protein (CRP)	1.89 mg/l	< 5 mg/l
Procalcitonin	0.03 ng/ml	< 0.5 ng/ml
LDH	327 IU/l	135-225 IU/l
Ferritin	326 mcg/l	21-326 mcg/l
Allbumin	32 g/L	35-52 g/L
Bilirubin total	4.1 micromol/L	< 17 micromol/L
Bilirubin direct	1.8 micromol/L	< 5 micromol/L
Alkaline phosphate	212 IU/L	40-129 IU/L
Aspartate aminotransferase (AST)	54 IU/L	< 40 IU/L
Alanine aminotransferase (ALT)	25 IU/L	< 41 IU/L
Complete blood count (CBC), not remarkable except for lymphopenia	1.85 x10 ^9/L	4-13.5 x10 ^9/L
Echocardiogram (Echo)	Normal	
Sputum culture	Pan-sensitive Klebsiella pneumonia	
Urine culture	No growth	
Blood culture	No growth	
PCR every 48-72 hour	Positive	

During the entire admission period, the patient was awake, alert, afebrile, hemodynamically stable, well-perfused, and maintained normal renal function; however, he required PICU admission due to increased oxygen requirement. Two days after initiating Remdesivir, weaning of oxygen started, and the patient maintained 100% saturation on 6L HFNC and FiO2 21%. On day five of Remdesivir, the patient was weaned from HFNC to a nasal cannula (NC). The patient maintained an oxygen saturation of 100% on 3L NC and FiO2 of 21%; then, he remained on 2L for four days until was off oxygen the day after stopping Remdesivir. After completing 10 days of Remdesivir and antibiotics, the patient was transferred out of the PICU to the regular pediatric COVID ward. Laboratory features after completing Remdesivir are shown below (Table 5).

**Table 5:** Laboratory features after completing 10 days of Remdesivir Arrows as a function of difference from the baseline before 10 days of Remdesivir: ↑ Value increased, ↓ Value decreased, ↔ Value remained same

Laboratory feature	Result	Normal result
↓AST	50 IU/L	< 41 IU/L
↔ALT	25 IU/L	< 40 IU/L
↑Direct bilirubin	3 micromol/L	< 5 micromol/L
↑LDH	336 IU/l	135-225 IU/l
↑Ferritin	714 mcg/l	21-326 mcg/l
PCR	Positive	

## Discussion

Remdesivir is only administered to children with COVID-19 following the National Guideline when there is evidence of active infection and positive PCR results [2]. Remdesivir is available in the market in the form of lyophilized powder and an injectable solution. Both Remdesivir formulations contain sulfobutylether beta-cyclodextrin sodium (SBECD). SBECD is known as a vehicle that is primarily eliminated through the kidneys.

Each 100 mg vial of Remdesivir lyophilized powder includes 3 g of sulfobutylether beta-cyclodextrin sodium SBECD, but each 100 mg/20 mL vial of Remdesivir the injectable solution contains 6 g of SBECD. 

A patient who receives a loading dosage of Remdesivir 200 mg would receive 6 g to 12 g of SBECD, depending on the formulation. Six to 12 grams of SBECD are regarded as being within the safety threshold in patients with normal renal function [8]. However, SBECD buildup in patients with renal insufficiency may lead to toxicities of the liver and the kidneys. Therefore, because lyophilized powder formulation includes less SBECD, it is preferable in patients with renal impairment [4].

Patients with an estimated glomerular filtration rate (eGFR) of less than 50 mL/min were nevertheless removed from some clinical trials since both Remdesivir formulations include SBECD, and those with an eGFR of less than 30 mL/min were also excluded in other investigations [4]. SBECD effects in young children whose kidneys are still growing and under the age of two are unknown, according to the manufacturer [9]. Furthermore, SBECD is dialyzable as a four-hour dialysis session may eliminate 46% of it [8].

There is no formal safety or pharmacokinetic data available for patients with kidney impairment, eGFR less than 30 mL/minute, or who are on renal replacement therapy [8]. However, for hospitalized pediatric patients who have an urgent or growing requirement for supplemental oxygen, the use of Remdesivir within the framework of a clinical study is advised, if possible [8].

Remdesivir was used in COVID-19 patients with renal impairment and eGFR 30 mL/min, even though it is not advised due to a lack of data [8]. However, it was used as the benefits outweigh the risks in some patients, and significant toxicity, when used for a short period of therapy (e.g., five to 10 days), is unlikely [8]. The use of Remdesivir in hospitalized COVID-19 patients with an eGFR below 30 mL/min was evaluated in two observational studies, and neither study found any appreciable differences in the incidences of adverse effects or acute kidney injury among patients with an eGFR below 30 mL/min and those with an estimated eGFR below 30 mL/min [4]. Renal function testing; however, is advised before and throughout Remdesivir therapy and is clinically required in both normal and impaired kidney function patients [4].

GS-441524 is the prominent metabolite of Remdesivir that is found to be elevated in cases of end-stage kidney disease [8]. In a patient known to be on intermittent hemodialysis and a five-day course of Remdesivir, GS-441524 was found to be elevated, and the hemodialysis prevented its accumulation and reduced the concentration by 50% [8]. In another observational study of three patients with end-stage kidney disease on hemodialysis who received Remdesivir for five days, elevated concentrations of GS-441524 were reported. However, hemodialysis reduced the concentration by 45%-49% [8]. 

Due to its low molecular weight (602.58 g/mol) and 87.9% protein binding, Remdesivir is eliminated by CRRT. As a result, in CRRT, greater doses may be necessary [10]. While medication lipophilicity and protein-bound rely on dose adjustment for patients receiving ECMO therapy [9]. Drugs with higher logP values are thought to have higher lipophilicity, which is determined by logP. Due to their increased solubility in the ECMO circuits compared to hydrophilic medicines, lipophilic pharmaceuticals tend to get bound [11].

Despite equal lipophilicity, medicines with high protein binding are lost in the ECMO circuit as a result of drug sequestration [4]. Remdesivir was categorized as being significantly eliminated using ECMO based on the computed LogP of the active medication and protein-bound Remdesivir. Standard dose regimens ought to be recommended going forward up to the time that pharmacokinetic and clinical data are available [10].

Remdesivir was utilized in three former preterm infants at Leicester Children's Hospital, according to a literature review [12]. SARS-CoV-2 RNA was detected in all three infants, and they all required oxygen and ventilator support. Blood cultures and tests for other respiratory viruses were both negative in all three infants. However, all neonates had higher levels of C-reactive protein, lactate dehydrogenase, ferritin, D-dimer, and NT pro-BNP. The first patient was born at 31 weeks and initially showed up six weeks later; at that time, the patient weighed 2.5 kg. The second and third neonates were delivered at 33 weeks and later presented at 2.5 weeks and five weeks of age, respectively. The third patient was 2.8 kg at the time of presentation, while the second neonate weighed 1.9 kg [12].

Remdesivir was administered at a dose of 1.25 mg/kg between days two and five after being started at a dose of 2.5 mg/kg on day one. A local recommendation was created considering the patient's age and weight. Two of the patients were extubated on day three of Remdesivir treatment, and the third patient was extubated on day two of Remdesivir therapy, indicating that the use of Remdesivir shortened the time to extubation. In two cases, no notable side effects were observed. However, after finishing the Remdesivir term, one patient who had a threefold increase in aspartate transaminase, AST (maximum 162 IU/L), returned to normal. All three individuals were discharged to their homes and have continued to be healthy. Remdesivir use in ex-preterm infants who weighed less than 3.5 kg was successful and eventful, according to the study's findings [12].

Laboratory and clinical studies were carried out on our two patients (cases 1 and 2) who were hospitalized and started on Remdesivir at SKMC PICU following the recommendations of the national criteria established by UAE COVID-19. Remdesivir was administered as both patients had positive PCR results and showed signs of elevated oxygen demand. Remdesivir lyophilized powder for injection was the formulation of choice in both cases because it has a better renal profile and contains less SBECD [9].

The dose recommendations and modifications were done with input from the PICU MDT and the on-call clinical pharmacist. A loading dose of 5 mg/kg/dose on day one and 2.5 mg/kg/dose once daily was recommended by the clinical pharmacist. Although the patient was 3.1 kg and on CRRT, the clinical pharmacist chose to administer Remdesivir at full loading and maintenance doses for case 1. When Remdesivir was started, the patient was already receiving ECMO and CRRT; ECMO and CRRT are known to remove Remdesivir from the system and reduce its buildup. In the second example, the patient, who weighed 1.69 kg, received a complete loading dosage and a maintenance dose because the patient was maintaining good urine output, had a normal renal function, and was putting up increasing oxygen effort.

Besides, the decision of giving full loading and maintenance doses for both patients was based on drug handling and administration as well. Weight-based dosing in low-weight patients (3.1 kg and 1.69 kg) can produce small volumes that may prove difficult to administer. The multidisciplinary team decided to give both patients a course of 10- days of Remdesivir. However, it was used only for eight days in case 1 as the PCR turned negative after two days of Remdesivir treatment and the patient’s condition improved significantly. In case 2, the patient completed 10 days of Remdesivir therapy as the PCR was still positive despite clinical condition improvement in the high-risk patient.

The most significant adverse reactions reported post-Remdesivir use were bradycardia, hepatic effects, and infusion reactions [6]. The mechanism by which Remdesivir is causing bradycardia is unknown. Nevertheless, it has been suggested that the active metabolite of Remdesivir may slow sinoatrial node automaticity due to its similarity with adenosine triphosphate causing bradycardia [8]. Whereas the effect on the liver was mild to moderate reversible transaminase elevations, which resolve upon discontinuation, including increased serum alanine aminotransferase and increased serum aspartate aminotransferase. However, it is unclear if these effects are drug-related or related to SARS-CoV-2 [8].

As per the manufacturer, Remdesivir is not recommended in patients with ALT≥ 5 upper normal range and is to be administered if the benefit outweighs the risk if ALT is elevated but less than five upper normal range [12]. There are no dosage adjustments provided in the manufacturer's labeling in case of hepatic impairment. Treatment needs to be discontinued if ALT > 10 times the upper normal range or ALT elevation in the presence of signs or symptoms of liver inflammation [8,9].

Remdesivir administration has been associated with hypersensitivity events, including anaphylaxis and infusion-related responses. To avoid hypersensitivity or infusion-related responses, the physician may consider slower infusion rates (maximum infusion time of up to 120 minutes) [8]. Remdesivir was infused over 60 minutes in both cases admitted in SKMC PICU. The total volume of the loading dose in case 1 was 12 ml, and the maintenance dose's total volume was 7.5 ml. In case 2, the final volume of the loading dose was 8.5 ml, and the volume of the maintenance dose was 4 ml. No infusion reaction or hypersensitivity was documented in both cases admitted to SKMC post-Remdesivir infusion. No elevation in AST, ALT, and bilirubin was noted during and after the Remdesivir course of therapy. Furthermore, no hepatic inflammation signs and symptoms were reported during and post-Remdesivir treatment. Compassionate use of Remdesivir for a short duration (5-10 days) in patients who weigh 3 kg or less and are renally impaired on renal replacement therapy appears to be safe and tolerable.

## Conclusions

Compassionate use of Remdesivir for a short duration (5-10 days) in patients who weigh 3 kg or less are renally impaired, and those on renal replacement therapy appear to be safe and tolerable. There were no Remdesivir-related adverse events noted during or post-therapy in the two cases reported. Moreover, clinical improvement, measurement of efficacy, and safety will require further randomized, placebo-controlled trials.
